# Development and Characterisation of a Novel NF-*κ*B Reporter Cell Line for Investigation of Neuroinflammation

**DOI:** 10.1155/2017/6209865

**Published:** 2017-07-16

**Authors:** Marie-Theres Zeuner, Thomas Vallance, Sakthivel Vaiyapuri, Graeme S. Cottrell, Darius Widera

**Affiliations:** ^1^Stem Cell Biology and Regenerative Medicine, School of Pharmacy, University of Reading, Reading RG6 6AP, UK; ^2^School of Pharmacy, University of Reading, Reading RG6 6AP, UK; ^3^Cellular and Molecular Neuroscience, School of Pharmacy, University of Reading, Reading RG6 6AP, UK

## Abstract

Aberrant activation of the transcription factor NF-*κ*B, as well as uncontrolled inflammation, has been linked to autoimmune diseases, development and progression of cancer, and neurological disorders like Alzheimer's disease. Reporter cell lines are a valuable state-of-the art tool for comparative analysis of in vitro drug screening. However, a reporter cell line for the investigation of NF-*κ*B-driven neuroinflammation has not been available. Thus, we developed a stable neural NF-*κ*B-reporter cell line to assess the potency of proinflammatory molecules and peptides, as well as anti-inflammatory pharmaceuticals. We used lentivirus to transduce the glioma cell line U251-MG with a tandem NF-*κ*B reporter construct containing GFP and *firefly* luciferase allowing an assessment of NF-*κ*B activity via fluorescence microscopy, flow cytometry, and luminometry. We observed a robust activation of NF-*κ*B after exposure of the reporter cell line to tumour necrosis factor alpha (TNF*α*) and amyloid-*β* peptide [1-42] as well as to LPS derived from *Salmonella minnesota* and *Escherichia coli*. Finally, we demonstrate that the U251-NF-*κ*B-GFP-Luc reporter cells can be used for assessing the anti-inflammatory potential of pharmaceutical compounds using Bay11-7082 and IMD0354. In summary, our newly generated cell line is a robust and cost-efficient tool to study pro- and anti-inflammatory potential of drugs and biologics in neural cells.

## 1. Introduction

The inducible transcription factor nuclear-factor “kappa-light-chain-enhancer” of activated B-cells (NF-*κ*B) is a key regulator of inflammation [1]. The NF-*κ*B protein family consists of the DNA-binding subunits p65 (RelA), RelB, c-Rel, p50, and p52 [2, 3], which bind to consensus elements on DNA (*κ*B-elements/binding sites with the sequence 5′GGGACTTTCC3′). In its inactive form, NF-*κ*B hetero- or homodimers reside in the cytoplasm bound to the inhibitory I*κ*B protein which prevents nuclear translocation of the complex. Upon activation of the signalling cascade, the inhibitor of NF-*κ*B (I*κ*B) kinase (IKK) complex consisting of IKK*α*, IKK*β*, and NF-*κ*B essential modulator (NEMO) is activated. IKK complex in turn phosphorylates inhibitory I*κ*B proteins, for example, I*κ*B*α*, I*κ*B*β*, or I*κ*B*ε* [4–6]. This phosphorylation at serine residues (e.g., serine 32 and 36 for I*κ*B*α*) promotes polyubiquitination and proteasomal degradation of I*κ*Bs [7]. The liberated NF-*κ*B dimers translocate into the nucleus and bind to their consensus elements and modulate the transcription of target genes [7]. This canonical NF-*κ*B signalling cascade can be activated by pathogen-associated molecular patterns (PAMPs) or damage-associated molecular patterns (DAMPs) (mediated by Toll-like receptor 4 (TLR4)), proinflammatory cytokines, and other external stimuli including UV-irradiation [8, 9].

Chronic inflammation and ongoing activation of NF-*κ*B is linked to a variety of diseases including inflammatory bowel disease [10], chronic obstructive pulmonary disease (COPD) [11], and neuropathologies like Alzheimer's disease [12] and epilepsy [13]. In addition, it has been shown that NF-*κ*B is involved in the development and progression of cancer [14]. In this regard, NF-*κ*B directly regulates cancer cell proliferation by upregulation of G1 cyclins, particularly cyclin D1 [15, 16]. Moreover, NF-*κ*B positively modulates expression levels of several chemokines (e.g., MCP-1) [17], proangiogenic factors, for example, vascular endothelial growth factor (VEGF) [18], and cellular inhibitors of apoptosis [19].

The human cell line U251-MG was isolated from a 75-year-old male Caucasian patient by explant technique from brain tissue of a grade III-IV malignant astrocytoma tumour at the Wallenberg laboratory in Uppsala (Sweden) in 1973 [20–22]. Nonclonal U251-MG cells are highly heterogeneous and contain differentiated cells and cancer stem cell-like cells [23]. The U251 cell line represents an ideal tool to study inflammatory signalling pathways. Indeed, this cell line has been used previously to study inflammatory responses in vitro [24–27]. In contrast to many standard cell lines, for example, HEK 293 which do not express TLRs, U251 cells are characterised by the expression of TLRs (except TLR2) [26], TNF receptor 1 (TNFR) [28], and interleukin receptors [27]. Thus, these cells are suitable for studying proinflammatory signals, particularly those proposed as endogenous TLR4 ligands, without overexpression artefacts.

In this study, we describe the development and characterisation of a U251-derived cell line stably transduced with a lentiviral NF-*κ*B-driven tandem reporter encoding for GFP and luciferase. We validated the cell line using established proinflammatory molecules including TNF*α* and bacterial lipopolysaccharides (LPS). Using this U251-NF-κB-GFP-Luc cell line, the NF-*κ*B activating properties of proposed endogenous TLR4-ligands (amyloid-*β* peptide [1-42] or high mobility group box 1 (HMGB1) and LPS chemotypes) were evaluated. Moreover, by using our reporter cell line, the anti-inflammatory potential and in vitro toxicity of anti-inflammatory drugs and biopharmaceuticals can be investigated.

## 2. Materials and Methods

### 2.1. Cell Culture

All cell lines were cultivated in normal cultivation medium (Dulbecco's Modified Eagle's Medium (DMEM) High Glucose (Sigma-Aldrich Company Ltd., Dorset, UK), 1% L-glutamine (200 mM, Sigma-Aldrich), 10% heat-inactivated fetal calf serum (FCS, lot:126K3398, Sigma-Aldrich)) in the absence of antibiotics and antimycotics in a humidified incubator (Thermo Fisher Scientific, Loughborough, UK) at 37°C and 5% CO_2_ unless stated otherwise. Cells were provided with fresh medium every 2-3 days and passaged at 90% confluency.

### 2.2. Lentiviral Transduction

HEK293-FT cells were cultivated with normal cultivation medium containing polyethylenimine (PEI, 0.1%). Medium was discarded after 16 h, and cells were incubated in normal cultivation medium containing 25 *μ*M chloroquine. Transfection was performed using 2 M CaCl_2_, TE-buffer, and 2x HBS supplemented with lentiviral package plasmids (6 *μ*g pCMV-VSV.G and 15 *μ*g pCMV-dR8.91) and 20 *μ*g pGreenFire-NF-*κ*B-Puro (System Biosciences, Palo Alto, CA, USA) vectors. After 16 h, medium was discarded and the virus particles were harvested after 24 h in normal cultivation medium. The virus containing medium was centrifuged at 1985*g* for 10 min and filtered through a 0.45 *μ*m filter (VWR International Ltd., Lutterworth, UK), prior to centrifugation (20000*g*, 2 h 15 min, 4°C). The supernatant was discarded, and the virus particles were resuspended in DMEM containing 1% L-glutamine and stored at 4°C. For lentiviral transduction of U251 cells, the medium was replaced with the prewarmed virus particle solution mixed with 10% FCS and incubated for 8 h at 37°C and 5% CO_2_ before normal cultivation medium was added. After 2 passages, cells were selected using 5 *μ*g/ml puromycin (Apollo Scientific, Bredbury, UK). After 2 weeks, 12 single clones were generated using limited dilution under normal culture conditions. Finally, 4 clones were analysed in activity assays. The clone showing the highest dynamics of luciferase activity in response to TNF*α* was used for all experiments.

### 2.3. Stimulation of Cells

For TNF*α* stimulation, cells were (unless otherwise indicated) treated with 10 ng/ml human recombinant TNF*α* (PeproTech EC Ltd., London, UK, in 0.1% BSA (cell culture grade, Sigma-Aldrich) in PBS (Sigma-Aldrich)) for 24 h. For immunocytochemical staining, cells were treated with TNF*α* for 15 min prior to fixation. All cells treated with TLR4 ligands (peptides and LPS) or NF-*κ*B inhibitors were serum starved for 4 h before stimulation for 48 h with normal cultivation medium comprising respective ligands: 1 *μ*g/ml (unless stated otherwise) ultrapure LPS derived from *Salmonella minnesota* (ultrapure *S. minnesota* R595 in endotoxin-free water, InvivoGen, Toulouse, France) or *Escherichia coli* (ultrapure *E. coli* LPS in endotoxin-free water, InvivoGen); 0.1–100 *μ*g/ml fibrinogen from human plasma (50–70%, Sigma-Aldrich, dissolved in DMEM High Glucose and sterile filtered); 50–1000 ng/ml high mobility group box 1 (human recombinant, carrier-free HMGB1, BioLegend Ltd., London, UK, dissolved in 0.1% BSA in PBS); 0.001–10 *μ*M amyloid-*β* peptide [1-42] (A*β*, rPeptide, Suffolk, UK, prepared in DMEM High Glucose) or heated A*β* (stock solution boiled at 90°C for 45 min); and 0.5–2.5 *μ*M Bay11-7082 (Sigma-Aldrich, in DMSO (cell culture grade, Apollo Scientific)) or IMD0354 (10 mM, Sigma-Aldrich, in DMSO).

### 2.4. Immunocytochemistry

U251 cells were seeded on cell culture-treated coverslips in 12-well plates (Sarstedt, Leicester, UK) and treated with TNF*α* as described above. After 20 min fixation in 4% paraformaldehyde (Sigma-Aldrich), cells were permeabilised using PBS containing 0.02% Triton X-100 (Sigma-Aldrich) and 5% normal goat serum (Stratech Scientific Unit, Suffolk, UK) for 30 min. Cells were incubated with mouse anti-human p65 primary antibody (1 : 100 in PBS, sc8008, Santa Cruz Biotechnology Inc., Santa Cruz, CA, USA) for 1.5 h at room temperature. Cells were exposed to secondary goat anti-mouse IgG conjugated to AlexaFluor555 (1 : 300 in PBS, Life Technologies Ltd., Paisley, UK) for 1 h at room temperature in the dark. Nuclear counterstaining was achieved using DAPI (1 : 2000 in PBS, Sigma-Aldrich).

### 2.5. Microscopy

Fluorescence microscopy of fixed samples was performed using AxioImager Epifluorence System (Carl Zeiss, Jena, Germany). Images of living cells (GFP and bright field images) were obtained using Nikon NIS Camera (Nikon, Surrey, UK) equipped with an A1 Inverted Epifluorence Microscope (Zeiss).

Image acquisition was performed using microscope-based analysis software (Axiovision4), and Fiji was used for pixel intensity measurement and further image processing [29].

### 2.6. Transfection of HEK293-MD2-CD14 and U251 cells

U251 cells (Cell Line Service, Eppelheim, Germany) or HEK293-MD2-CD14 cells (InvivoGen) were transfected with pRL-CMV (Promega Corporation, Southampton, UK), TK (NF-*κ*B_6_) LUC [30], TLR4-GFP (InvivoGen), or EGFP (after removal of endotoxins according to Ma et al. [31]) using Turbofect® Tranfection Reagent (Thermo Fisher Scientific). Transfection was assessed by expression of GFP using epifluorescence microscopy (A1 Inverted Epifluorescence Microscope, Carl Zeiss).

### 2.7. Luciferase Measurement

NF-*κ*B-dependent *firefly* luciferase activity and NF-*κ*B-independent *Renilla* luciferase activity were assessed using Dual-Luciferase® Reporter Assay System (Promega Corporation). Luciferase activity of U251-NF-*κ*B-GFP-Luc cells was analysed using *firefly* luciferase assay system (Promega Corporation), and all luciferase measurements were performed using a Lucy 1 microplate reader (Anthos Labtec, Salzburg, Austria).

### 2.8. Flow Cytometry

U251-NF-*κ*B-GFP-Luc cells were cultivated and treated for 24 h with TNF*α* or vehicle as described above. After Trypsin-EDTA (Sigma-Aldrich) treatment and centrifugation (300*g*, 10 min), cells were resuspended in PBS. GFP fluorescence intensity (10^5^ events per sample) was measured using BD Accuri™ C6 plus flow cytometer (BD Biosciences, USA). Data were analysed using FlowJo (FlowJo LLC, Ashland, USA).

### 2.9. MTT Assay

U251-NF-*κ*B-GFP-Luc cells were serum-deprived for 4 h and exposed to LPS derived from *E. coli* and *S. minnesota* (10^−2^, 10^−1^, 1, 10^1^, and 10^2^ *μ*g/ml) or left untreated for 68 h. MTT assays (Promega Corporation) were performed according to the manufacturer's guidelines, and readout was carried out using Lucy 1 microplate reader (Anthos Labtec).

### 2.10. Statistical Analysis

All statistical analyses were performed using GraphPad Prism software (GraphPad, La Jolla, CA, USA). Data were compared using either Student's *t*-test (two-tailed, confidence interval 95%) or one-way analysis of variance (ANOVA) with Bonferroni correction (CI 95%), where appropriate. At least 3 independent measurements were performed, and *p* < 0.05 was considered statistically significant. Luciferase and MTT data are presented as mean ± SEM, and pixel intensity measurements are presented as mean ± SD.

## 3. Results

### 3.1. The Inflammatory Response in U251 Cells Is Heterogeneous

**Figure 1:**
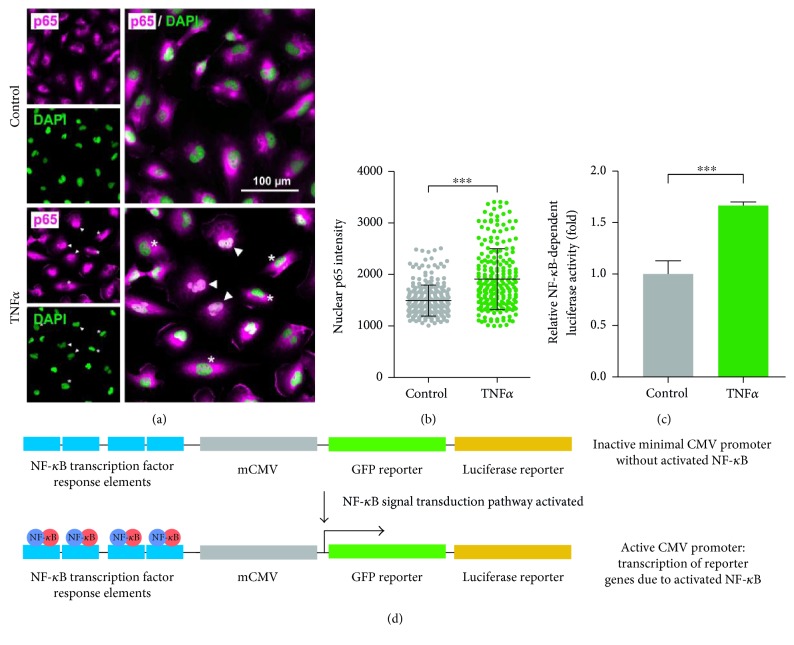
U251 cells respond heterogeneously to a proinflammatory trigger. (a) Immunocytochemistry of the NF-*κ*B subunit p65 (magenta) shows nuclear translocation of NF-*κ*B after stimulation with TNF*α* for 15 min (arrowheads). Note the heterogeneous response in p65 nuclear translocation in U251 cells (asterisk). Scale bar: 100 *μ*m. (b) Quantification of nuclear p65 intensity showed significant increase of nuclear translocated NF-*κ*B in TNF*α*-treated U251 cells compared to untreated U251-cells. Data are presented as mean ± SD from three different measurements (merged), compared using Student's *t*-test (unpaired, two-tailed, CI 95%). ^∗∗∗^*p* < 0.001. (c) Increased relative luciferase activity was observed in U251 cells transiently transfected with a NF-*κ*B-dependent luciferase reporter. Mean ± SEM from three different experiments are shown, analysed using unpaired Student's *t*-test (two-tailed, CI 95%). ^∗∗∗^*p* < 0.001. (d) Schematic display of the lentiviral NF-*κ*B-dependent luciferase reporter. NF-*κ*B activation leads to binding of NF-*κ*B to respective response elements, enhancing GFP and luciferase reporter gene expression through activation of the minimal CMV promoter.

To assess TNF*α*-driven nuclear translocation of NF-*κ*B in U251 cells, p65 subunit localization was assessed by immunofluorescence, followed by acquisition of its fluorescence intensity in the nucleus (Figures 1(a) and 1(b)). Treatment with TNF*α* resulted in significantly increased nuclear p65 fluorescence compared to unstimulated cells (Figure 1(a) lower panel, Figure 1(b)). Notably, the TNF*α*-induced nuclear translocation in the nonclonal U251 population is heterogeneous with a highly responsive subpopulation (Figure 1(a), arrowheads), cells with intermediate levels of nuclear p65, and cells without nuclear translocation (Figure 1(a), asterisk).

Similarly, subpopulation of cells spontaneously translocating p65 into the nucleus was identified in nonstimulated cells (control, Figure 1(b)). In parallel experiments, U251 cells were cotransfected with a NF-*κ*B-dependent luciferase reporter and a constitutively active *Renilla* luciferase construct (transfection control) (Figure 1(c)). Significantly increased NF-*κ*B-dependent luciferase activity was observed after 24 h exposure to TNF*α* compared to the control (Figure 1(c)).

### 3.2. U251-NF-*κ*B-GFP-Luc Cell Line Combines Visual Readout and Quantification of NF-*κ*B Activation

**Figure 2:**
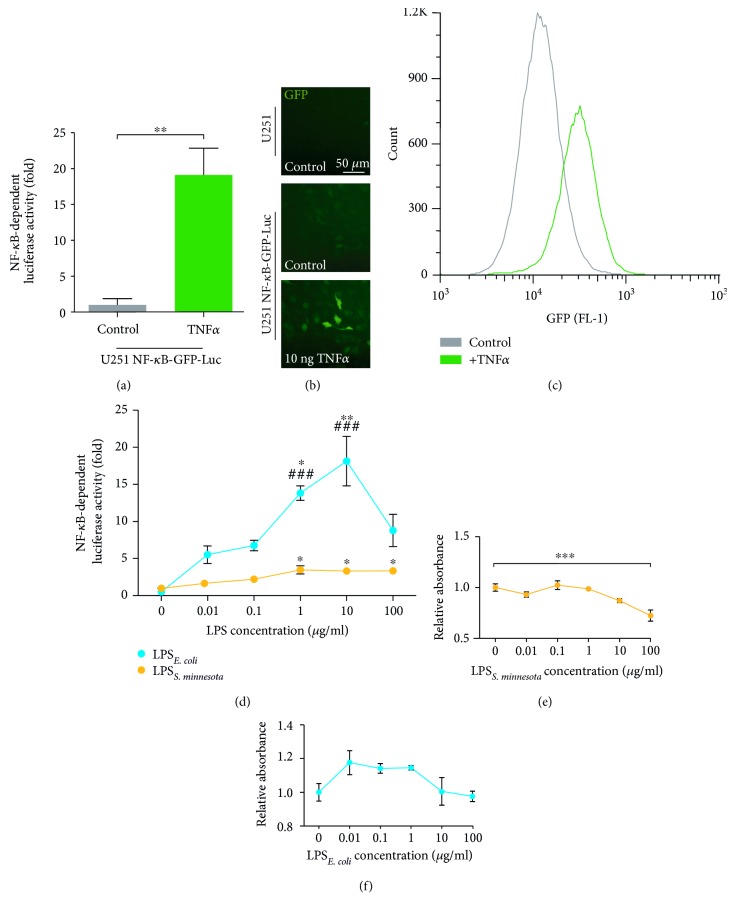
GFP and luciferase expression is increased in U251-NF*κ*B-GFP-Luc cells after activation of NF-*κ*B signalling. (a) Stimulation with 10 ng/ml TNF*α* for 24 h significantly increases NF-*κ*B-dependent luciferase activity in U251-NF-*κ*B-GFP-Luc cells. Mean ± SEM from three independent experiments are shown, compared using Student's *t*-test (unpaired, two-tailed, CI 95%). ^∗∗^*p* < 0.01. (b) Fluorescence microscopy and (c) flow cytometry was applied to visualize the increase of GFP expression in U251-NF-*κ*B-GFP-Luc cells after 24 h stimulation with 10 ng/ml TNF*α*. Scale bar: 100 *μ*m. (d) U251-NF-*κ*B-GFP-Luc cells were triggered for 24 h with different concentrations of LPS derived from *E. coli* (LPS_*E. coli*,_ blue) or *S. minnesota* (LPS_*S. minnesota*,_ yellow) (0.01, 0.1, 1, 10, and 100 *μ*g/ml), and NF-*κ*B-dependent luciferase activity was analysed. A significantly higher luciferase activity was shown using *E. coli* LPS compared to *S. minnesota* LPS. (e) U251-NF-*κ*B-GFP-Luc cells were treated with different concentrations of *S. minnesota* LPS or (f) *E. coli* LPS for 3 days before analysis using an MTT assay. Note that 100 *μ*g/ml *S. minnesota* LPS significantly decreased cell viability. The presented values are mean ± SEM from three different experiments, analysed using ANOVA with Bonferroni correction (^∗^*p* < 0.05, ^∗∗^*p* < 0.01, and ^∗∗∗^*p* < 0.001 was considered significant, CI 95%) to compare between concentrations or an unpaired Student's *t*-test (^###^*p* < 0.001, two-tailed, CI 95%) to compare the two chemotypes at a specific concentration.

In order to combine both a visual and quantifiable readout without the influence of population heterogeneity, we lentivirally transduced U251 cells using the pGreenFire-NF-*κ*B-Puro plasmid (Figure 1(d)). When stimulated with different concentrations of TNF*α*, clone A1 (U251-NF-*κ*B-GFP-Luc-A1) revealed the highest levels of luciferase luminescence and thus it was used in further experiments (Figure 2(a)).

Next, U251-NF-*κ*B-GFP-Luc-A1 cells were exposed to TNF*α*, followed by an assessment of GFP fluorescence using fluorescence microscopy and flow cytometry (Figures 2(b) and 2(c)). No GFP fluorescence was detected in control U251 cells, whereas low basal signal was observed in unstimulated U251-NF-*κ*B-GFP-Luc-A1 cells (Figure 2(b)). Notably, TNF*α* treatment significantly increased the levels of GFP in U251-NF-*κ*B-GFP-Luc-A1 cells compared to untreated reporter cells.

### 3.3. Biased Agonism Is Detectable in U251-NF-*κ*B-GFP-Luc Cells

In a recent study, we reported that LPS chemotypes with different acetylation patterns mediate differential levels of NF-*κ*B activation in U251 cells [26]. To test if the newly developed cell line has sufficient sensitivity to detect broad ranges of LPS concentrations and ligand-dependent activation of NF-*κ*B, we applied various concentrations of LPS derived from either *S. minnesota* or *E. coli* and analysed the NF-*κ*B-dependent luciferase bioluminescence. In accordance with our previous findings obtained in untransduced U251 cells [26], we were able to detect concentration-dependent changes in NF-*κ*B activation for both LPS chemotypes (Figure 2(d)). In addition, the assay was also able to detect ligand-dependent differences in NF-*κ*B activation (*E. coli* versus *S. minnesota* LPS) (Figure 2(d)). Next, we investigated the viability of U251-NF-*κ*B-GFP-Luc cells using MTT assays. Cells were exposed to variable concentrations of *E. coli* or *S. minnesota* LPS, and viability was assessed after 3 days. We observed significantly decreased viability of the cells when treated with 100 *μ*g/ml *S. minnesota* LPS after 3 days compared to nontreated cells (Figure 2(e)). In contrast, concentration of *E. coli* LPS had no effect on the viability of U251-NF-*κ*B-GFP-Luc cells (Figure 2(f)).

### 3.4. U251-NF-*κ*B-GFP-Luc Cells Can Be Used in Screening for Endogenous TLR4-Ligands

NF-*κ*B is an important readout in TLR4 signalling, and we demonstrated that this reporter cell line can be used to distinguish between ligand-dependent and concentration-dependent effects of TLR4 ligands (Figure 2(d)). Therefore, we investigated NF-*κ*B activation in response to the potential endogenous TLR4 ligands fibrinogen, HMGB1, and amyloid-*β*.

**Figure 3:**
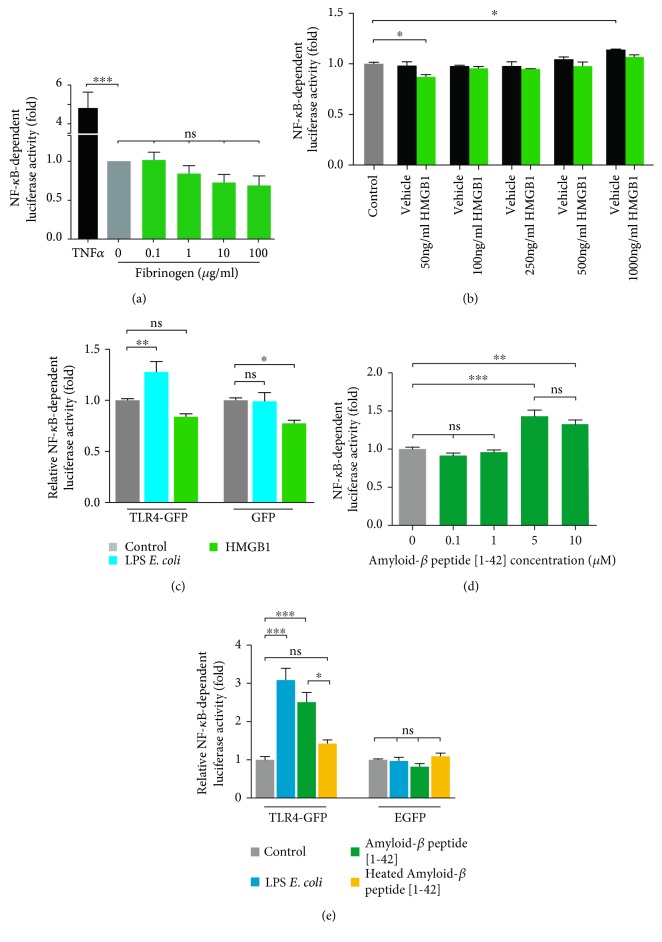
Proposed endogenous TLR4-ligands can be tested using U251-NF-*κ*B-GFP-Luc cells (a). No changes in NF-*κ*B-dependent luciferase activity were observed using 0.1, 1, 10, or 100 *μ*g/ml fibrinogen. (b) 50 ng/ml high mobility group box 1 (HMGB1) led to a slight decrease whereas vehicle (0.1% cell culture grade BSA in PBS) induced an increase in luciferase activity in U251-NF-*κ*B-GFP-Luc cells. (c) Evaluation of TLR4-dependence in response to HMGB1 using HEK293-MD2-CD14 cells transfected with a transient NF-*κ*B-luciferase reporter and GFP or TLR4-GFP. 50 *μ*M HMGB1 led to a significant decrease in cells transfected with GFP only. *E. coli* LPS: positive control. Control: nontreated. (d) 5 and 10 *μ*M, but not 0.1 and 1 *μ*M amyloid-*β*-peptide [1-42], significantly increased NF-*κ*B-dependent luciferase in the U251 reporter cells. (e) HEK293-MD2-CD14 were cotransfected with a transient NF-*κ*B-luciferase reporter and GFP or TLR4-GFP. 5 *μ*M amyloid-*β*-peptide [1-42], but not heated amyloid-*β* (control for LPS contamination), significantly increased luciferase activity. *E. coli* LPS: positive control. Control: nontreated. Data are presented as mean ± SEM from at least 3 independent experiments. ^∗^*p* < 0.05, ^∗∗^*p* < 0.01, and ^∗∗∗^*p* < 0.001 were considered significant, ANOVA with Bonferroni correction, CI 95%.

No activation of NF-*κ*B was observed when U251-NF-*κ*B-GFP-Luc cells were treated with fibrinogen (Figure 3(a)). Next, we examined NF-*κ*B-driven luciferase activity after exposure of the reporter cells to HMGB1. A significantly decreased luciferase activity was measured when cells were stimulated with 50 ng/ml HMGB1, compared to unstimulated controls (Figure 3(b)). At higher concentrations, no effect of HMGB1 treatment was detectable; however, a significant increase was observed in cells treated with the respective amount of vehicle alone (0.1% BSA in PBS) (Figure 3(c)).

To further investigate the effect of HMGB1, we used a TLR-deficient cell background. HEK293 cells stably expressing the TLR4 coreceptors MD2 and CD14 were transfected with a transient NF-*κ*B-dependent luciferase reporter construct, a *Renilla* luciferase transfection control, and TLR4-GFP or GFP, respectively. Thus, it was expected that TLR4 ligands would promote an increase in luciferase activity in TLR4-GFP cells but not in cells expressing GFP alone. Here, 50 ng/ml HMGB1 also led to decreased NF-*κ*B-dependent luciferase expression in TLR4-deficient cells (Figure 3(d)).

Amyloid-*β* [1-42] peptide promotes TLR4-mediated activation of NF-κB, often in association with TLR2 [32]. To investigate if amyloid-*β* [1-42] can activate NF-*κ*B in the absence of TLR2, we exposed U251-NF-κB-GFP-Luc cells to increasing concentrations of amyloid-*β*. Amyloid-*β* significantly increased NF-κB-dependent luciferase activity (Figure 3(e)). HEK293-MD2-CD14 cells were transfected with respective luciferase reporters and either TLR4-GFP or only GFP. Stimulation with 5 *μ*M amyloid-*β* or 1 *μ*g/ml *E. coli* LPS did not result in NF-*κ*B activation in the absence of TLR4. However, in the presence of TLR4, amyloid-*β* significantly increased luciferase activity, comparable to *E. coli* LPS (Figure 3(f)). The amyloid-*β* peptide used here was an *E. coli*-derived recombinant protein, and thus LPS contamination could not be excluded. As LPS is not heat sensitive [33], we heat-inactivated the amyloid-*β* peptide (hA*β*) for 45 min at 90*°*C, which has been reported to denaturate occurring oligomers and interfere with biological activity [34]. The relative NF-*κ*B-dependent luciferase activity in the hA*β* was significantly lower compared to the native amyloid-*β* peptide and not significantly different from the untreated control (Figure 3(f)).

### 3.5. U251-NF-*κ*B-GFP-Luc Cells Can Be Used as a Screening System for Anti-Inflammatory Compounds

**Figure 4:**
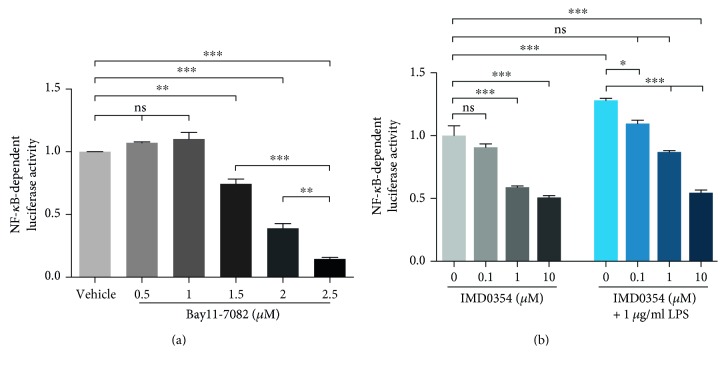
U251-NF-*κ*B-GFP-Luc cells are suitable for testing the anti-inflammatory potential of drugs (a). The NF-*κ*B-inhibitor Bay11-7082 significantly reduced NF-*κ*B-dependent luciferase activity at concentrations higher than 1.5 *μ*M. Vehicle: DMSO. (b) The IKK*β* inhibitor IMD0354 diminished NF-*κ*B-dependent luciferase activity at various concentrations (0.1, 1, and 10 *μ*M) both in the presence or absence of 1 *μ*g/ml *E. coli* LPS. Data are presented as mean ± SEM from at least 3 independent experiments. ^∗^*p* < 0.05, ^∗∗^*p* < 0.01, and ^∗∗∗^*p* < 0.001 were considered significant, ANOVA with Bonferroni correction, CI 95%.

To evaluate whether the moderate basal NF-*κ*B activity of U251-NF-*κ*B-GFP-Luc cells is helpful in detection of anti-inflammatory molecules or drugs, we first tested a range of concentrations of Bay11-7082, a general IKK inhibitor (poorly selective for IKK1 or IKK2) proposed as a potential compound for glioma treatment [35] without an additional proinflammatory trigger. We detected a significant decrease of basal NF-*κ*B activity at concentrations higher than 1.5 *μ*M compared to DMSO-treated (vehicle) cells (Figure 4(a)).

In addition, the function of the IKK*β* is essential for NF-*κ*B-activation, as it phosphorylates the cytosolic inhibitor of NF-*κ*B, I*κ*B. Moreover, IKK*β* is part of NF-*κ*B activation in the MyD88-dependent signalling pathway triggered by *E. coli* LPS. We therefore examined IMD0354, an IKK*β* inhibitor, in the absence and presence of *E. coli* LPS. 1 *μ*M and 10 *μ*M IMD0354 significantly decreased the NF-*κ*B-dependent luciferase activity in U251-NF-*κ*B-GFP-Luc cells. When simultaneously stimulated with *E. coli* LPS, 0.1 *μ*M IMD0354 slightly reduced NF-*κ*B activity; however, 1 *μ*M and 10 *μ*M IMD0354 resulted in significant reduction of LPS-mediated activation of NF-*κ*B (Figure 4(b)). In sum, the basal NF-*κ*B activity of U251-NF-*κ*B-GFP-Luc cells is very valuable in detection of anti-inflammatory drugs.

## 4. Discussion

NF-*κ*B plays a pivotal role in the development and homeostasis of the immune system, epithelium, and skeletal system (reviewed in [36]) as well as the central nervous system (reviewed in [37]). Due to this central physiological role, its dysregulation is associated with a range of pathological conditions, including autoinflammatory neurodegenerative disorders and cancer.

In this study, we describe a new NF-*κ*B reporter cell line, suitable as a screening system to investigate pro- and anti-inflammatory biologicals and pharmaceutical compounds.

Several NF-*κ*B reporter cells have been generated to date. The most common cells used for the generation of NF-*κ*B reporter cell lines are HEK293 cells stably transfected with a NF-*κ*B luciferase reporter (System Biosciences (NF-*κ*B/293/GFP-Luc™) or SEAP (secreted embryonic alkaline phosphatase, Invivogen (HEK-Blue™ Null cells)). However, a common drawback of HEK293-based reporter cells is the absence of endogenously expressed TLRs. Therefore, these receptors must be (stably or transiently) overexpressed resulting in potential overexpression artefacts (reviewed in [38]).

Other available NF-*κ*B reporter cell lines include a colon carcinoma cell line (HCT116, Invivogen) and lung carcinoma cell line (A549, Invivogen). Both of these are dual reporter cell lines, generated for the simultaneous investigation of the activation of interferon regulatory factor (IRF3/7) [39] and NF-*κ*B. These reporter systems are very valuable when studying TLR4-mediated signalling. However, these cells are not particularly useful when studying neuroinflammation.

The macrophage THP-1-NF-*κ*B-Lucia cell line (Invivogen) can also be used for investigation of proinflammatory signalling mediated by TLR4. Importantly, immunomodulation and macrophage polarization (M1, M2) can be studied using these cells. However, THP-1-NF-*κ*B-Lucia cells require a nonstandard luciferase substrate (for Lucia luciferase) and additional chemicals for cultivation (zeocin, normocin).

Until now, no NF-*κ*B reporter cell line has been generated from a glioblastoma cell line. Constitutively active NF-*κ*B is associated with invasive behaviour, increased proliferation, and inflammation of various cancers including glioblastoma [40]. In accordance to that, we observed a basal NF-*κ*B-driven GFP expression in U251-NF-*κ*B-GFP-Luc cells (Figures 1, 2(a), 2(b), and 2(c)), indicating constitutively active NF-*κ*B. When triggered with the proinflammatory cytokine, TNF*α*, both luciferase expression and GFP fluorescence intensity were increased (Figures 2(b) and 2(c)). The advantage of our cell line compared to others mentioned previously is that NF-*κ*B activity can be monitored at both population (luciferase) and single cell (GFP) level.

Previously, we have demonstrated that the response of U251 cells after exposure to bacterial LPS depends on the chemotype of the LPS [26]. TLR4-mediated signalling can lead to two different signalling pathways, dependent on the intracellular adapter protein. The MyD88-dependent pathway leads to an early NF-*κ*B activation, whereas the MyD88-independent pathway leads to activation of interferon regulatory factor 3 (IRF3), and also to a later activation of NF-*κ*B. As NF-*κ*B is the common denominator in signalling mediated by TLR4, we investigated if biased signalling can be examined using U251-NF-*κ*B-GFP-Luc cells. We successfully demonstrated that our newly generated reporter cell line is suitable to investigate biased TLR4 signalling, in this case in response to *E. coli* or *S. minnesota* LPS (Figure 2(d)).

Although pathogen-associated inflammation has been intensely studied, sterile (nonseptic) inflammation due to endogenous molecules is not completely understood and even debated. Several studies suggest an involvement of TLRs in the occurrence of nonseptic inflammation. In this regard, most endogenous ligands studied until now seem to activate NF-*κ*B signalling via TLR4 [41], particularly fibrinogen [42] and HMGB1 [43, 44]. U251 cells endogenously express proteins involved in the TLR4 signalling pathway and do not need to be transfected [25, 26]. In U251-NF-*κ*B-GFP-Luc cells, fibrinogen had no effect on NF-*κ*B-activation whereas HMGB1 seemed to decrease the activity of NF-*κ*B. The effect of HMGB1 was further verified using the commonly used HEK293-MD2-CD14 cells. Here, HMGB1 decreased the luciferase activity in both cells transfected with respective luciferase reporters and GFP and TLR4-GFP, showing that HMGB1 might not be a specific ligand for TLR4. Although we observed a slight increase in NF-*κ*B-dependent luciferase activity with increasing HMGB1 concentrations in our reporter cells (Figure 3(b)), it is very likely that this response was due to the vehicle used as recommended by the manufacturer (0.1% BSA in PBS). Notably, we were able to detect an increase in luciferase activity when U251-NF-*κ*B-GFP-Luc cells were stimulated with increasing amounts of 0.1% BSA in the cultivation medium (Figure 3(b)).

Recently, TLR4 and TLR2 have been shown to play a significant role in neuroinflammation occurring during Alzheimer's disease [12, 32]. By stimulating TLR2-deficient U251-NF-*κ*B-GFP-Luc cells and TLR4-transfected HEK293-MD2-CD14 cells with the amyloid-*β* peptide [1-42], we confirmed that amyloid-*β* signals through TLR4, leading to NF-*κ*B activation in our NF-*κ*B reporter cells (Figure 3(c)). Tsan and Gao [45] recently proposed that positive responses after exposure to endogenous protein ligands are due to LPS contaminations of the peptide solutions. They demonstrated that heat inactivation can be used to detect LPS contaminations in recombinant proteins produced in *E. coli* [45]. Heat-inactivated amyloid beta peptide did not promote a NF-*κ*B-dependent response in our reporter cell line, indicating that our observations are not due to contaminations with endotoxins (Figure 3(e)).

We also tested if our reporter cells can be used as a pharmacological tool to identify anti-inflammatory compounds (Figure 4). In the present study, we also showed that our cell line can be used to investigate NF-*κ*B inhibitors, as shown as using previously characterised inhibitors of the NF-*κ*B signalling pathway, Bay11-7082, and IMD0354. Bay11-7082 has already been applied to U251 cells by Wang et al. to demonstrate the importance of NF-*κ*B in the resistance to chemotherapy [46]. In our study, both inhibitors significantly reduced NF-*κ*B-dependent luciferase activity independently of the presence of LPS, pointing out the benefits of the basal NF-*κ*B activity when inhibitors are applied (Figure 4). Notably, stably transduced cells are known to undergo genotypic changes with increasing cultivation time even in the presence of selection antibiotics. Consequently, a prolonged cultivation of the U251-NF-*κ*B-GFP-Luc cells may result in a phenotypic shift and a potentially reduced responsiveness over time. In our hands, U251-NF-*κ*B-GFP-Luc cells were phenotypically stable for up to 10 passages. However, we recommend to prepare an adequate number of frozen stocks at early passages.

## 5. Conclusions

In this study, we described the generation and characterisation of a stable NF-*κ*B reporter cell line allowing the assessment of NF-*κ*B activity based on the expression of GFP and luciferase activity. As aberrant regulation of NF-*κ*B activation is associated with several diseases, for example, cancer, pharmacological tools for identification and cytotoxicity of NF-*κ*B-inhibiting molecules are of medical interest. Our reporter cell line can be used for investigation of proinflammatory molecules and peptides. Moreover, cytotoxicity and mode of action of anti-inflammatory pharmaceuticals can be investigated.
